# Supplementary material for: Interleaved single and bursting spiking resonance in neurons
Source: PLoS Comput Biol. 2025 May 22;21(5):e1013126. doi: 10.1371/journal.pcbi.1013126 (PMC12136421; doi:10.1371/journal.pcbi.1013126)
Supplement: S1 Text — Fig A. Ionic currents effect on neuronal response during sustained depolarization. Firing rate and coefficient of variation (CV), a measure the activity of the neuron and the presence of bursts, displayed as heatmaps spanned over different ionic current variation. A GKA vs. Input current. B GKM vs. Input current. C GfKCa vs. Input current. D GsKCa vs. Input current. Fig B. Ionic currents effect on neuronal response during oscillatory input. Firing rate and coefficient of variation (CV), a measure the activity of the neuron and the presence of bursts, displayed as heatmaps spanned over different frequency input variation. A GKA vs. Input current. B GKM vs. Input current. C GfKCa vs. Input current. D GsKCa vs. Input current. E Voltage traces of control vs after blocking M-type potassium current. Fig C. Time evolution of activation variables. A Voltage trace showing interleaved single spiking and bursting during oscillatory stimulation. Asterisks represent spike peaks. B. Time evolution of activation variables of IKDR, IKM and INaP. Note the different temporal scales. Fig D. Effect of noise on interleaved resonance. Default values for fixed conductance while varying the other are GNaP = 0.93, GKDR = 5.86, and Gh = 8.46. A1-2 Δf and ΔA for varying values of GNaP. B1-2 Δf and ΔA for varying values of GKDR. C1-2 Δf and ΔA for varying values of Gh. Colors mark the magnitude of noise (blue = low, orange = medium, green = high). Fig E. Somatic and dendritic traces under gamma and theta oscillations input. Somatic traces (top) and dendritic traces (bottom). Fig F. Intermediate GNaP and GKDR generates firing rate resonance even at low input amplitudes. Same simulation as in Fig 2, but for lower input amplitudes (2x). We have also raised the holding potential by to be closer to threshold for this experiment (offset amplitude: 2 μA/cm2) A Firing rate while varying stimulation frequency of an oscillatory input for GKDR = 5. B Firing rate while varying stimulation frequency of an [file pcbi.1013126.s001.docx]

**Supporting information for**

**Interleaved single and bursting spiking resonance in neurons**

Cesar C. Ceballos^1^, Nourdin Chadly^3,4^, Eric Lowet^3^, Rodrigo F. O. Pena^1,2, *^

^1^ Department of Biological Sciences, Florida Atlantic University, Jupiter, FL, USA
^2^ Stiles-Nicholson Brain Institute, Florida Atlantic University, Jupiter, FL, USA
^3^ Department of Neuroscience, Erasmus Medical Center, Rotterdam, Netherlands
^4^ Swammerdam Institute for Life Sciences, University of Amsterdam, Amsterdam, Netherlands

***** Corresponding author
E- mail: [penar@fau.edu](mailto:penar@fau.edu)

**Fig A.** **Ionic currents effect on neuronal response during sustained depolarization.** Firing rate and coefficient of variation (CV), a measure the activity of the neuron and the presence of bursts, displayed as heatmaps spanned over different ionic current variation. **A** *G*_KA_ vs. Input current. **B** *G*_KM_ vs. Input current. **C** *G_f_*_KCa_ vs. Input current. **D** *G_s_*_KCa_ vs. Input current.

**Fig B.** **Ionic currents effect on neuronal response during oscillatory input.** Firing rate and coefficient of variation (CV), a measure the activity of the neuron and the presence of bursts, displayed as heatmaps spanned over different frequency input variation. **A** *G*_KA_ vs. Input current. **B** *G*_KM_ vs. Input current. **C** *G_f_*_KCa_ vs. Input current. **D** *G_s_*_KCa_ vs. Input current. **E** Voltage traces of control vs after blocking M-type potassium current.

**Fig C.** **Time evolution of activation variables. A** Voltage trace showing interleaved single spiking and bursting during oscillatory stimulation. Asterisks represent spike peaks. **B.** Time evolution of activation variables of *I*_KDR_, *I*_KM_ and *I*_NaP_. Note the different temporal scales.

**Fig D.** **Effect of noise on interleaved resonance.** Default values for fixed conductance while varying the other are *G*_NaP_ = 0.93, *G*_KDR_ = 5.86, and *G*_h_ = 8.46. **A1-2** $\Delta f$ and $\Delta A$ for varying values of *G*_NaP_. **B1-2** $\Delta f$ and $\Delta A$ for varying values of *G*_KDR._ **C1-2** $\Delta f$ and $\Delta A$ for varying values of *G*_h_. Colors mark the magnitude of noise (blue = low, orange = medium, green = high).

**Fig E.** **Somatic and dendritic traces under gamma and theta oscillations input.** Somatic traces (top) and dendritic traces (bottom).

**Fig F. Intermediate *G*_NaP_ and *G*_KDR_ generates firing rate resonance even at low input amplitudes.** Same simulation as in Fig 2, but for lower input amplitudes (2x). We have also raised the holding potential by to be closer to threshold for this experiment (offset amplitude: 2 $\mu$A/cm^2^**) A** Firing rate while varying stimulation frequency of an oscillatory input for *G*_KDR_ = 5. **B** Firing rate while varying stimulation frequency of an oscillatory input for *G*_NaP_ = 0.35.
